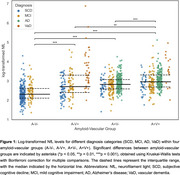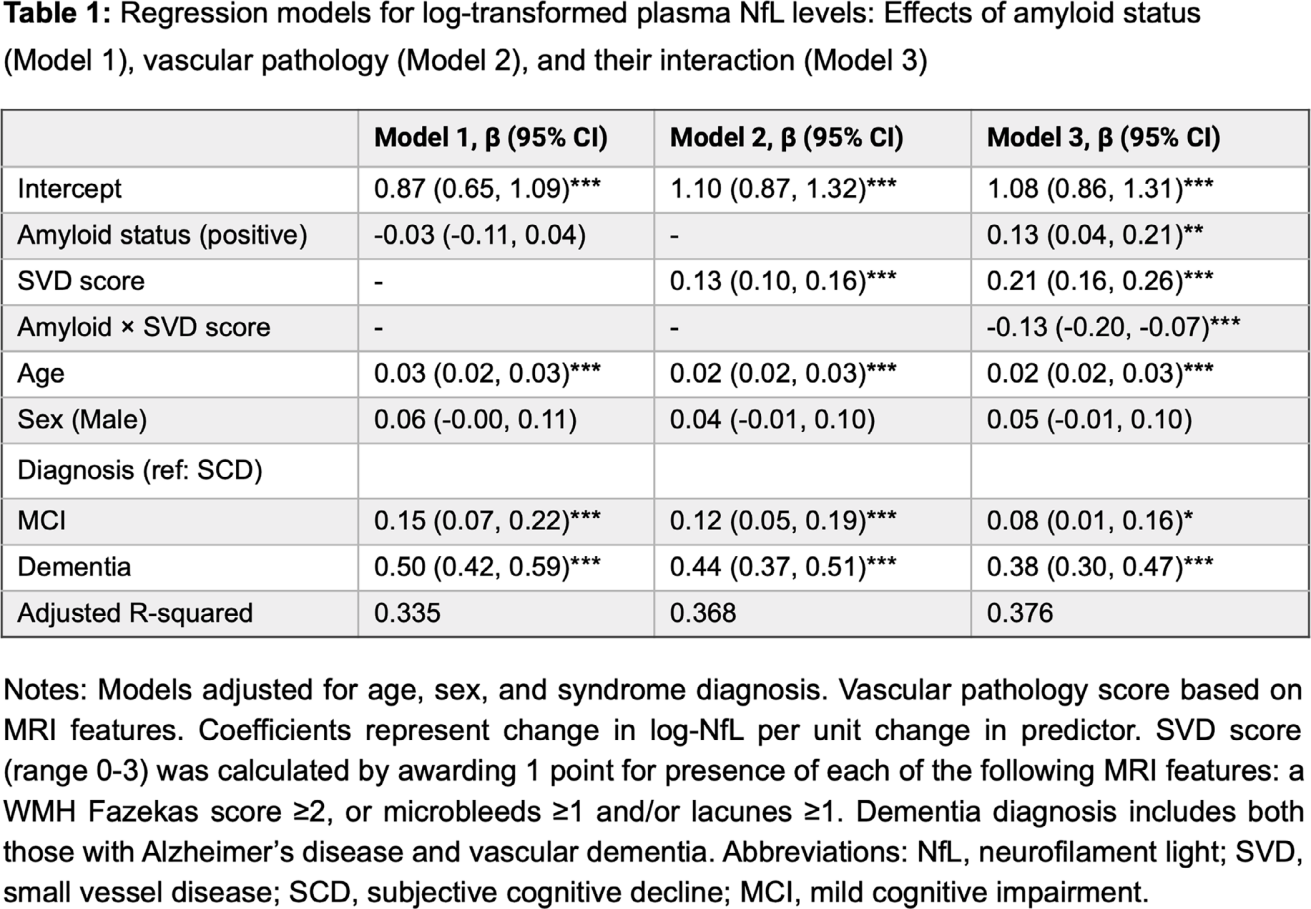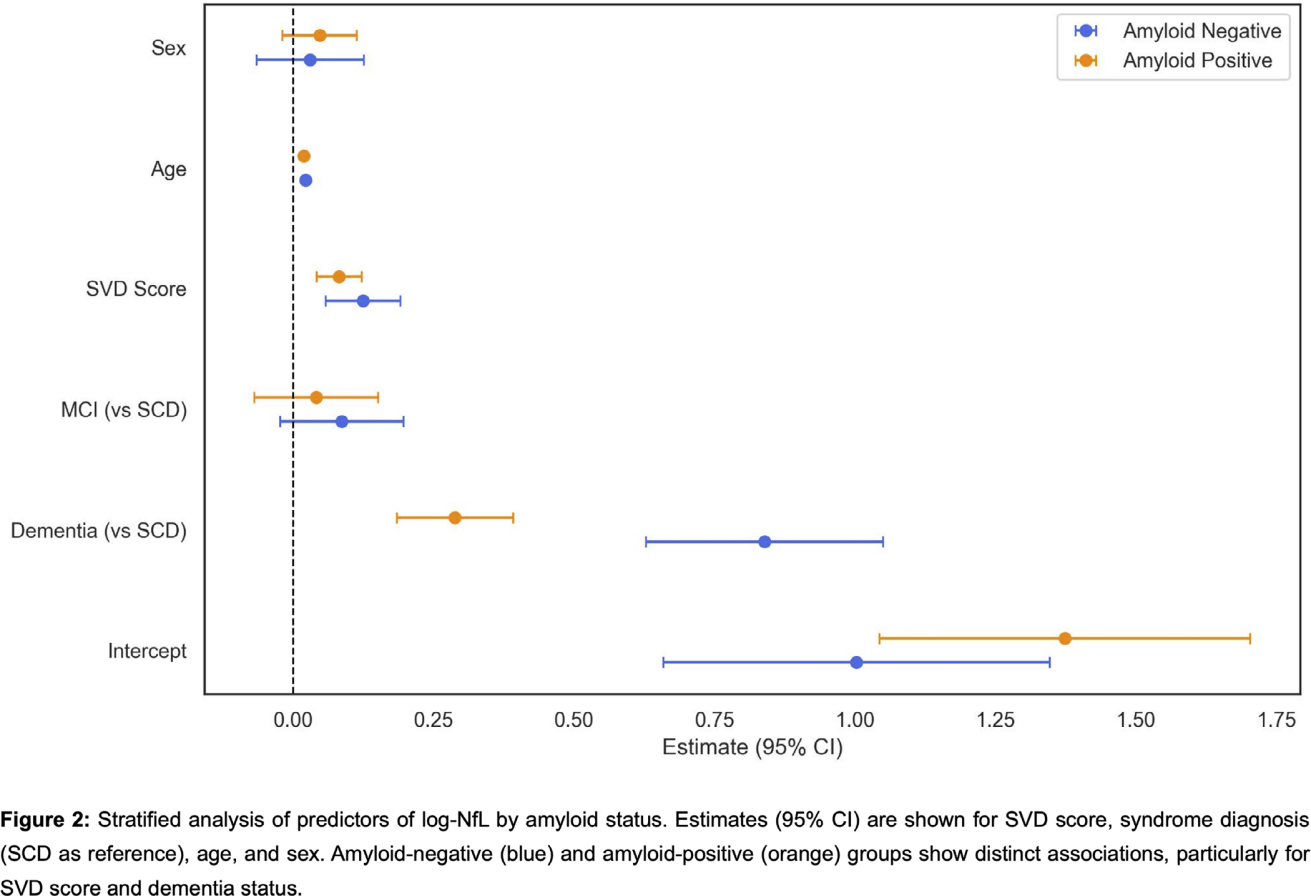# Interplay between Alzheimer and vascular pathology as determinants of plasma neurofilament light in a memory clinic population

**DOI:** 10.1002/alz70856_104832

**Published:** 2026-01-07

**Authors:** Michelle C. Barboure, Inge M.W. Verberk, Sinthujah Vigneswaran, Frederik Barkhof, Elsmarieke van de Giessen, Geert Jan Biessels, Charlotte E. Teunissen, Wiesje M. van der Flier, Argonde C. van Harten

**Affiliations:** ^1^ Alzheimer Center Amsterdam, Department of Neurology, Amsterdam UMC, location VUmc, Amsterdam, Netherlands; ^2^ Neurochemistry Laboratory, Department of Laboratory Medicine, Amsterdam UMC, Vrije Universiteit Amsterdam, Amsterdam Neuroscience, Amsterdam, Netherlands; ^3^ Alzheimer Center, Department of Neurology, Amsterdam UMC, Vrije Universiteit Amsterdam, Amsterdam Neuroscience, Amsterdam, Netherlands; ^4^ Amsterdam UMC, location VUmc, Amsterdam, Noord‐Holland, Netherlands; ^5^ Amsterdam Neuroscience, Brain Imaging, Amsterdam, Netherlands; ^6^ Department of Radiology & Nuclear Medicine, Amsterdam UMC, Amsterdam, Netherlands; ^7^ Department of Neurology, UMC Utrecht Brain Center, University Medical Center Utrecht, Utrecht, Netherlands

## Abstract

**Background:**

Plasma neurofilament light (NfL) is increasingly used as a general marker for neurodegeneration in memory clinics to aid differential diagnosis. However, interpreting its levels in the presence of vascular pathology remains unclear. We investigated the individual and combined effects of Alzheimer's and vascular pathologies on plasma NfL in individuals across the cognitive spectrum.

**Method:**

Cross‐sectional data from 1099 individuals in the Amsterdam Dementia Cohort were analyzed, including participants with a clinical diagnosis of subjective cognitive decline (SCD, *n* = 371), mild cognitive impairment (MCI, *n* = 326), Alzheimer's disease (AD, *n* = 347), and vascular dementia (VaD, *n* = 55). NfL was measured using SIMOA (Quanterix) and log‐transformed. Amyloid pathology was defined by positive CSF/PET biomarkers. Vascular pathology was defined as having a small vessel disease (SVD) score ≥1 (range 0‐3), with one point given for each feature present: white matter hyperintensities (Fazekas score ≥2), microbleeds (≥1), and/or lacunes (≥1). Participants were classified by vascular (V) and amyloid (A) status: A‐V‐ (*n* = 293), A‐V+ (*n* = 173), A+V‐ (*n* = 325), A+V+ (*n* = 308). Group differences were calculated using Kruskal‐Wallis with Bonferroni correction. Multivariate regression analyses investigated the effects of amyloid and vascular pathology on NfL levels, adjusting for age, sex, and syndrome diagnosis.

**Result:**

NfL levels increased stepwise from A‐V‐ to A+V+ groups, suggesting an additive effect of amyloid and vascular pathologies (Figure 1). Subsequent regression analysis revealed SVD score as a significant predictor of NfL levels, while amyloid status was not (Model 1 and 2, Table 1). An interaction term revealed that amyloid status moderated the effect of SVD score on NfL levels (Model 3). Stratified analysis showed higher baseline NfL levels in amyloid‐positive individuals, with SVD score remaining a significant predictor in both groups. While included as a covariate, dementia diagnosis emerged as a strong determinant of NfL levels, particularly in amyloid‐negative individuals (Figure 2). SVD score explained more variance in amyloid‐negative (40%) than amyloid‐positive (21%) individuals.

**Conclusion:**

Vascular burden influences NfL levels, particularly in amyloid‐negative individuals. These results underscore the importance of considering multiple pathologies when interpreting NfL as a neurodegeneration biomarker in memory clinic settings.